# Unconventional implant strategy for patients with a limited interocclusal space in the posterior region: a case report

**DOI:** 10.1186/s12903-019-0907-1

**Published:** 2019-09-18

**Authors:** Yi Wang, Wen Cen, Jiang-Qin Huang, Hong-Wu Wei

**Affiliations:** Department of Oral Implantology, The Fourth Affiliated Hospital of Nanchang University, School of Medicine, Nanchang University, No. 133 Guang-Chang-Nan Road, Nanchang, China

**Keywords:** Implant-supported dental prosthesis, Locking-taper implants, Single-tooth implants

## Abstract

**Background:**

Implant prostheses require sufficient interocclusal space. In cases of limited interocclusal space, reducing or extracting over-erupted opposing teeth, orthodontic intrusion, or surgical reconstruction of the edentulous space are commonly used to restore the interocclusal space. However, there are disadvantages to these approaches.

**Case presentation:**

The present case report describes a patient with a limited interocclusal space managed using an unconventional implant strategy.

**Conclusions:**

The patient presented satisfactory outcomes without any signs of implant failure, suggesting that the unconventional implant treatment strategy is a useful option for patients with a limited interocclusal space in the posterior region. This unconventional implant surgery provides a minimally invasive treatment alternative.

## Background

Dental implants are surgically placed into the jaw bones to restore missing teeth [1]. Conventionally, dental implants have been used successfully in clinical practice with a convincing ten-year survival rate (95%) [2, 3]. Adequate interocclusal space is necessary for proper implantation of a functional dental prosthesis. According to Misch [4], the interocclusal space should be at least 8–12 mm in vertical distance. However, unrestored edentulous space due to loss of posterior teeth results in random movement of adjacent teeth, which compromises the ability to place an implant. In such cases, common prosthodontic treatments using conventional dental implants is challenging for clinicians [5].

In cases of reduced interocclusal space, implantologists have several options to optimize the space available for the implant. However, these options may further prolong or complicate the treatment. These options include: reducing the over-erupted opposing teeth [6] (associated risk - loss of healthy enamel and endodontic treatment may be necessary); surgical reconstruction of the edentulous space such as with posterior maxillary segmental osteotomy [7, 8] (associated risk - complications of an additional surgery such as risk of wound infection); orthodontic intervention to intrude the extruded teeth [5, 9] (associated risk - cost and prolonged treatment time); and using a screw-retained, implant-supported casted abutment with an integrated crown [10, 11] (associated risk - custom fabrication of abutment). These solutions also require removal of healthy tooth/bone structure and are expensive and time consuming. Additionally, surgical reconstruction involves invasive wound healing and potential surgical complications, which further increase cost and time. Hence, there is a need for a more straightforward and effective solution to manage patients with a reduced interocclusal space.

A number of studies have reported success using deep placement of locking-taper implants [12–14]. Thus, we proposed a new protocol based on the hypothesis that deeply placed locking-taper implants can effectively create upper space and lower the restorative platform of the implant supported restorations. We report one case with a limited interocclusal space in the posterior region and management using deep placement of locking-taper implants.

## Case presentation

This study was conducted following the ethical guidelines defined by the World Medical Association as described in the Declaration of Helsinki released in 2013. Patients meeting the selection criteria (Table 1) underwent the unconventional dental implant treatment.

**Table 1:** Patients’ selection criteria

Inclusion criteria	Exclusion Criteria
● Good state of general health	● Presence of Oral pathology such as ulcer, premalignant lesions
● Well maintained oral hygiene	● History of alcoholism/smoking
● The height of the mandible must be > 11 mm for deep installation of implant*	● Severe periodontitis and bone loss
	● The height of interocclusal space is more 5 mm
● Patients’ understanding of deviation from standard care and the risk of implant failure	● History of radiotherapy in the head and neck region

**Fig. 1:**
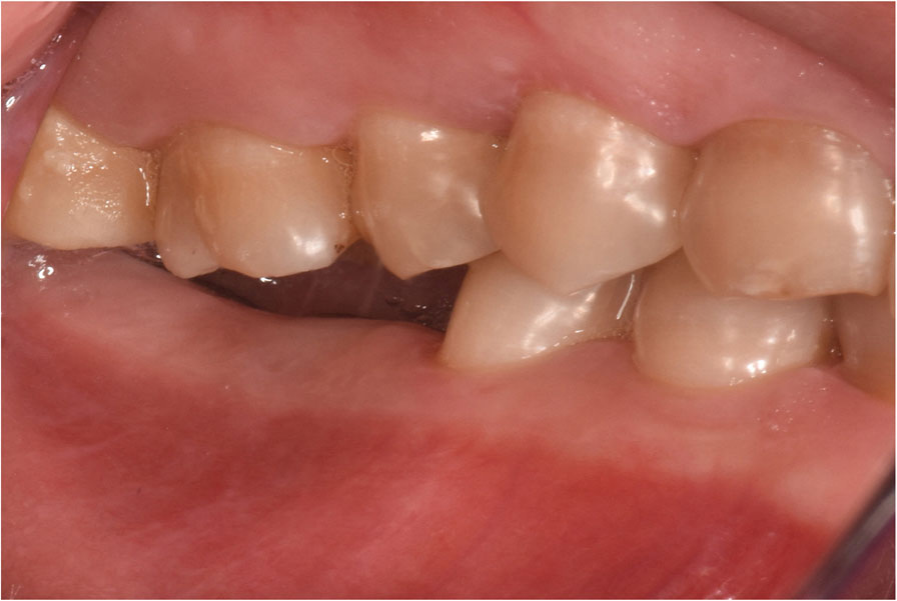
Preoperative clinical presentation showing reduced interocclusal space in the centric occlusion

In 2016, a 40-year old female patient visited the Department of Oral Implantology at The Fourth Affiliated Hospital of Nanchang University to replace her missing 1st and 2nd molars in the right lower jaw. The intraoral examination revealed that the patient had right mandible vertical dimensions of 3 mm and 1 mm at the 1st and 2nd molars, respectively, as shown in Fig. 1. Consequently, there was a lack of vertical dimension required to replace the right mandibular molars. The remaining teeth showed some tetracycline stains without any other dental abnormalities. The radiographic evaluation by cone beam computed tomography (CBCT) showed sufficient bone mass for dental implant placement.

Following complete clinical evaluation, the proposed treatment plan was comprised of restoring the lost interocclusal space and placement of two implants. Regaining the lost interocclusal space may lead to alternative solutions. The treatment plan and expected outcomes were explained to the patient as described below:

i. Prevailing care is essential while restoring vertical dimensions prior to implant placement.
ii. The treatment may be more complex.
iii. An easier alternative approach would be to place the locking-taper implants deeply to regain the native lost interocclusal space.
iv. Although promising outcomes have been reported for deeply placed locking-taper implants in the upper and lower jaws [12], there is no available standard protocol.
v. For any complication, substitution implants using the standard protocol will be offered to replace the deteriorating implants.

**Fig. 2:**
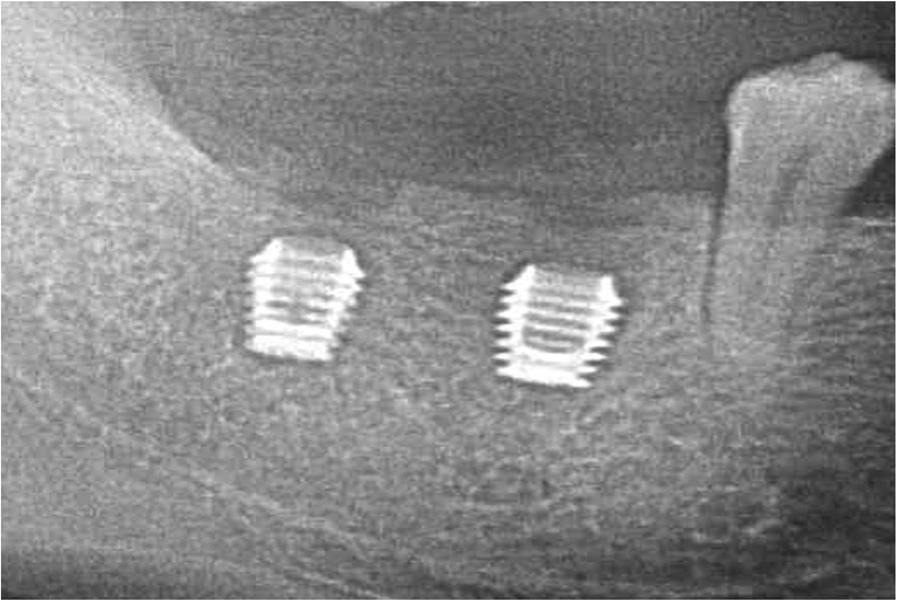
Deeply placed implants

**Fig. 3:**
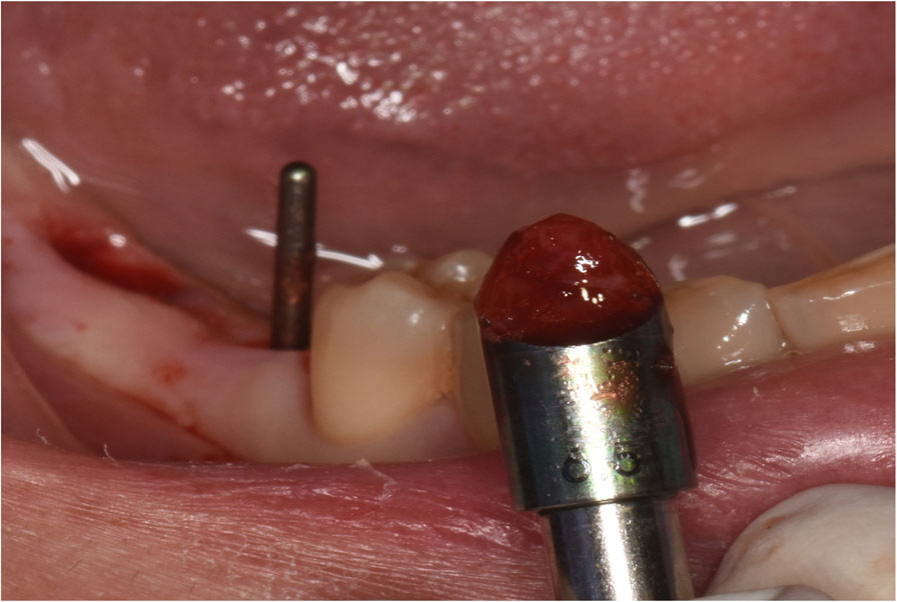
Removal of any residual bone with Sulcus Reamer

**Fig. 4:**
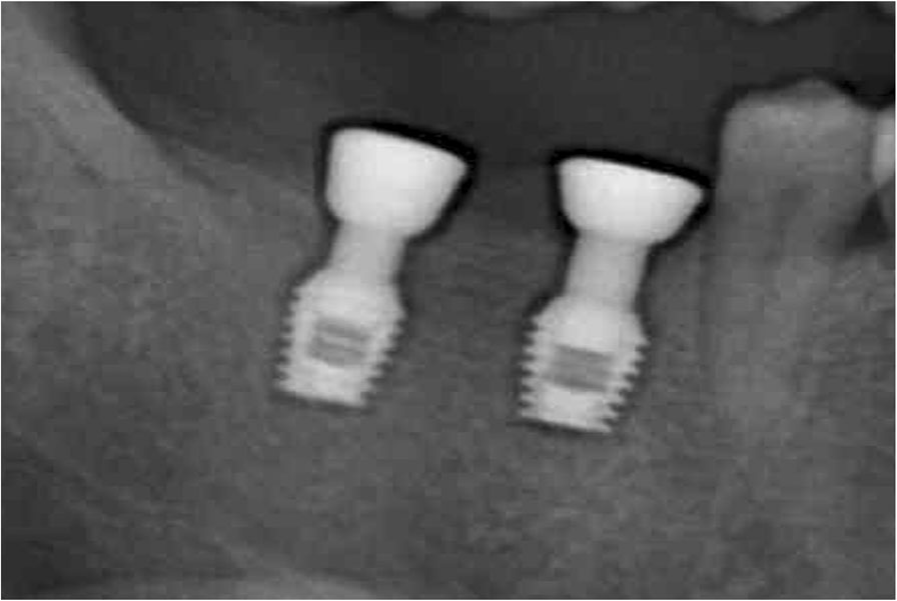
Radiographic view of healing abutments

The patient accepted the treatment plan using the locking-taper implants and signed the informed consent. The surgical procedure was performed under local anesthesia using articaine with adrenaline 1:100,000 (Pierrel, Milan, Italy). Two locking-taper implants were placed deeply (Bicon LLC, Boston, MA, USA), as shown in Fig. 2, using the drilling sequence recommended by the manufacturer. At the same time the patient was instructed to use chlorhexidine mouthwash (0.2% for 1 min, thrice daily) starting 3 days prior to the surgery and 1 week postoperatively. The patient received a standard antibiotic prophylaxis (amoxicillin, 2 g/day for 7 days) starting on the day of surgery. For the purpose of healing, submerged implants were left to recover for 5 months, followed by a second surgery to access the underlying implants and remove any residual bone using a Sulcus Reamer, as shown in Fig. 3. The healing abutments were placed and activated, as shown in Fig. 4**.**

**Fig. 5:**
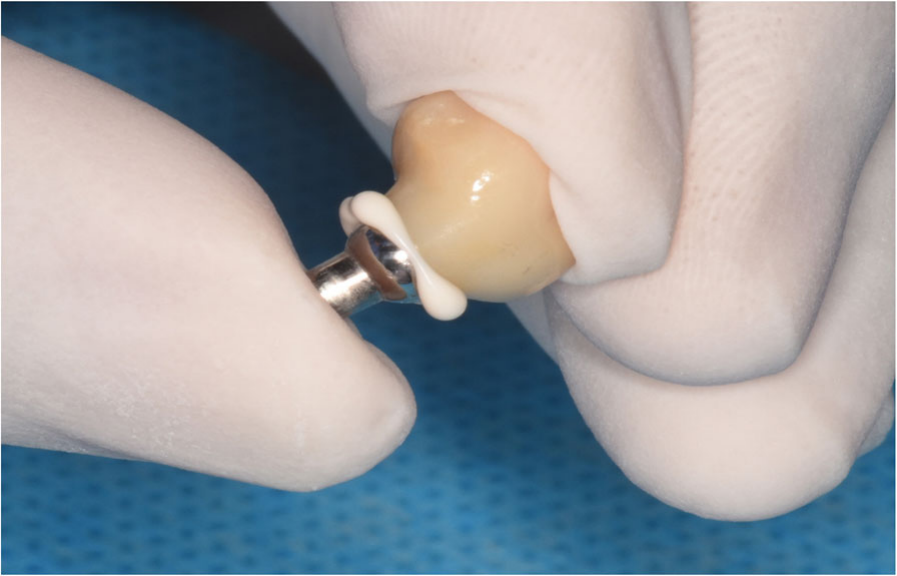
Extraoral cementation of the crown

**Fig. 6:**
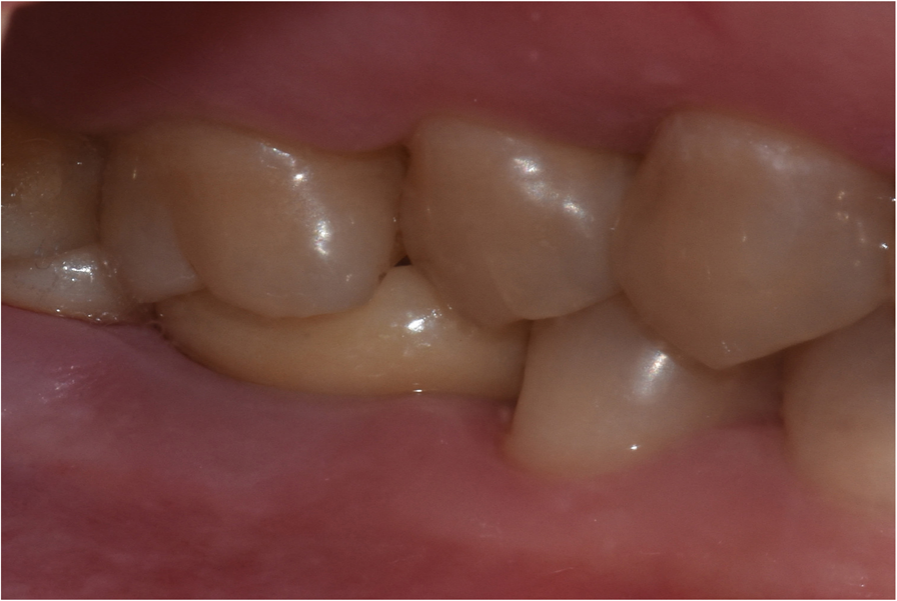
Clinical view of centric occlusion of the final restoration

**Fig. 7:**
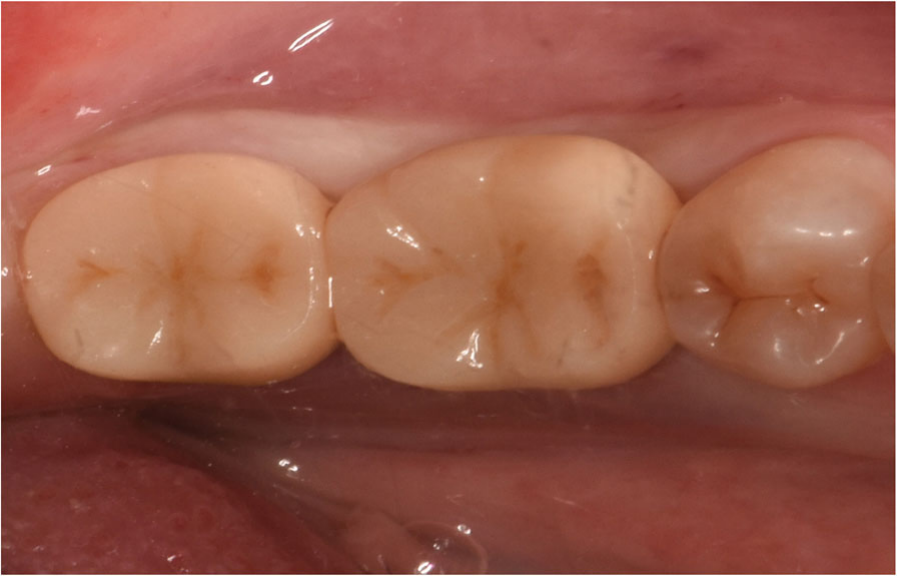
Clinical view of occlusal table the final restoration

At one month follow-up, the Express™ Vinyl Polysiloxane impression (3 M Espe Dental, Medizin, Germabny) was obtained for planning a prosthesis (crown with subgingival margins). After 1–2 weeks, we used the extraoral cementation technique [16] to cement the fabricated single porcelain crowns and restore the implants, as shown in Fig. 5. Following insertion, the centric occlusion was checked to remove any premature occlusal spot and achieve the proper occlusion, protrusion, and laterotrusion, as shown in Figs. 6 and 7.

Following the completion of treatment, the patient was recalled on a yearly basis for clinical and radiographic evaluation. At each follow-up visit, a dentist and a prosthodontist (prosthodontists were blind to the treatment plans) assessed the patient for changes in the marginal peri-implant bone level, mobility, and any other signs of clinical failure.

The current study followed the survival criteria listed by Albrektsson and Zarb [15] and are briefly described below:

i. Absence of pain, sensitivity, or any exudate
ii. No implant mobility or peri-implant radiolucency
iii. Bone-to-implant contact (DIB) < 1.5 mm following functional loading of 12 months, and no more than 0.2 mm/annum for the following years
iv. Absence of any kind of prosthetic complications

Radiographic evaluation was performed to rule out the presence of any radiolucency around the implant or any pathological bone reaction around the bone-implant interface.

**Fig. 8:**
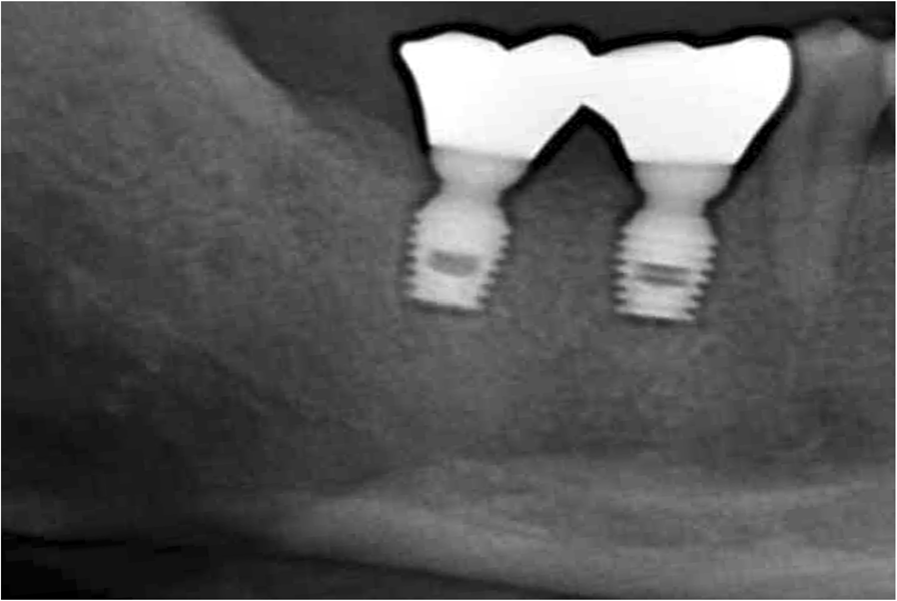
Radiographic view of follow-up 2 years after implants

In the current study, both implants placed in the patient healed physiologically without any complication during the scheduled follow-up period (up to 2 years). The patient reported suitable functioning of the implant prosthesis. Additionally, there were no unusual clinical or radiographic features associated with any of the inserted implants, as shown in Fig. 8.

## Discussion and conclusion

The present case investigated the deep placement of locking-taper implants for a patient with a decreased interocclusal space. The patient was followed for 2 years for radiographic and clinical assessment. The patient presented satisfactory outcomes without any signs of implant failure, suggesting that the unconventional implant treatment strategy is a useful option for patients with a limited interocclusal space in the posterior region.

To manage patients with limited interocclusal space in the posterior region, we presented a strategy that may be less invasive compared to other strategies. For example, the reduction of opposing extruded molars may lead to dentinal hypersensitivity, requiring endodontic treatment and additional crowns. Alternatively, the orthodontic intrusion of teeth is a technique sensitive procedure that takes a longer time and may not be suitable for most of patients. The surgical reconstruction, such as posterior maxillary segmental osteotomy, involves more trauma of the surgical procedure and associated complications such as wound infection and healing issues.

We have proposed an unconventional protocol for the prosthetic management of patients with a limited interocclusal space.

In the second-stage surgery, the bone superior to the dental implant can be removed using special instruments (Sulcus Reamer) to regain the interocclusal space. This procedure flattens out the bone around the implant (but does not flatten the bone level around the implant), regains the interocclusal space, and facilitates the placement of the dental implant-abutment. This approach of removing the bone superior to the dental implant has a number of advantages compared to conventional treatment. First, this approach is very simple and cost effective and can be offered to patients of all socioeconomic statuses. Second, the amount of bone removal in the second-stage is more accurate and minimally invasive compared to flattening the bone level before implantation. Third, patients can be protected from the hazards of invasive procedures, such as loss of dental or bone tissues and surgical trauma while gaining the interocclusal space using conventional approaches.

In the third-repair-stage, retaining methods for implant dentures used cemented retention. Compared to the screw retained implant, the cemented prosthesis has a number of advantages, such as comfort, aesthetics, and ease of handling. In contrast, the main disadvantage is localized irritation due to excess dental cement, which may lead to peri-implantitis [17]. In cases of subgingival crown margins, excessive cement may be compressed during cementation between the implant-abutment surface and peri-implant soft tissues; hence its removal is not easy [18]. In the present case study, the patient’s interocclusal space was reduced. In this situation, the restoration margins were placed subgingivally to increase the retention. Therefore, we used locking-taper implants utilizing the extraoral cementation technique [16], taking advantage of implant-abutment connection characteristics. The cementing process between the abutment and the crown was completed outside the mouth after the trial was completed. In addition to the above benefits, the unconventional method [16] facilitates easy removal of excess cement between the abutments and restorations, which reduces the risk of peri-implant disease. Consequently, regaining interocclusal space through this approach and using crowns with subgingival margins increases the height and retention area for better adhesion between the abutment and crown.

In the past, studies have suggested that malocclusion and occlusal interferences were the main factors in the development of temporomandibular disorders (TMD) [19]. However, more recent studies have shown no remarkable differences in relation to signs and symptoms of TMD among subjects with malocclusion and those with physiological occlusion [20, 21]. In the present study, no clinically relevant temporomandibular joint disorder was observed during the clinical follow-up. This may be attributed to an appropriate adaptation capacity of TMJ tissues that can compensate for small functional alterations created by the presence of the malocclusions [22]. However, further long-term clinical studies are needed to validate these findings.

Based on a 2 year follow-up period, the patient in the present study reported clinical success and functionality of the locking-taper implants. Neither of the two implants presented any radiographic or clinical signs of failure. Therefore, we suggest that the locking-taper implants can be selectively considered for patients with a limited interocclusal space. Further studies with prolonged follow-up time are required to evaluate the clinical success of this unconventional technique.

## Data Availability

The datasets generated and/or analyzed during the current study are not publicly available due to inconsistent language expression, but are available from the corresponding author upon reasonable request.

## Abbreviations

CBCT: Cone beam computed tomography
DIB: Bone-to-implant contact
TMD: Temporomandibular disorders
